# Comparison of measurement of the apical anatomy of maxillary central incisors using cone-beam computed tomography and conventional intraoral radiographs

**DOI:** 10.1186/s12903-026-07774-0

**Published:** 2026-02-09

**Authors:** Josephine Solgaard Henriksen, Eva Lauridsen, Simon Storgård Jensen, Tron Andre Darvann, Shumei Murakami, Tomomi Tsujimoto, Yuka Uchimoto, Nuno Vibe Hermann

**Affiliations:** 1 Pediatric Dentistry and Clinical Genetics, Department of Odontology, Faculty of Health and Medical Sciences, University of Copenhagen, Copenhagen, Denmark; 2 Department of Oral and Maxillofacial Surgery, Copenhagen University Hospital, Rigshospitalet, Copenhagen, Denmark; 3 Oral Surgery, Research Section for Oral Biology and Immunopathology, Department of Odontology, Faculty of Health and Medical Sciences, University of Copenhagen, and Department of Oral and Maxillofacial Surgery, Copenhagen University Hospital, Rigshospitalet, Copenhagen, Denmark; 4 3D-Craniofacial Image Research Laboratory, Copenhagen University Hospital, Rigshospitalet, Faculty of Health and Medical Sciences, University of Copenhagen, DTU informatics, Copenhagen, Denmark; 5 Department of Oral and Maxillofacial Radiology, Osaka University Graduate School of Dentistry, Osaka, Japan

**Keywords:** Apical foramen, Apical constrictor, Healing potential, Age, Incisors, Traumatic dental injuries

## Abstract

**Purpose:**

The aim of this study was to investigate the dimension of the apical foramen in maxillary incisors in relation to age as observed in conventional intra-oral periapical radiographs and Cone-Beam CT (CBCT). We hypothesized a discrepancy in apical foramen size between 2D radiographs and 3D CBCT, and that the foramen narrows with age.

**Materials and methods:**

Seventy patients with 87 anterior maxillary teeth were included. The shape, size and the area of the apical foramen were measured corresponding to the narrowest part of the canal system (the apical constrictor/minor foramen) on intraoral conventional X-rays and CBCT scans taken in close succession. A comparison between the two imaging methods was performed and related to the age of the patient, with statistical analyses including t-test, regression, and Bland-Altman plots. In addition, the area of the foramen was measured, and a shape index was calculated from the CBCT scans.

**Results:**

The area of the apical foramen measured in 3D using CBCT, decreased at a rate of -0.019 mm_2_ per month. The diameter of the foramen decreased at a rate of -0.014 mm per month (2D, mesiodistal dimension), -0.013 mm per month (3D, mesiodistal) and − 0.016 mm pr month (3D, faciolingual). The correlation between the mesiodistal diameter in 2D and 3D was fair with *R* = 0.60, and the correlation between mesiodistal and faciolingual diameter in 3D was high (*R* = 0.77). The mean shape index was found to be 0.992 ± 0.244.

**Conclusion:**

The present study found that area and diameter (mesiodistal and faciolingual directions) of the foramen, measured to be equivalent to the apical constrictor, decreased with age. The most common shape of the apical constriction was round. There was a significant difference between the diameter of the foramen measured on conventional periapical radiographs compared to the CBCT scans.

## Introduction

Numerous studies have investigated the root and canal morphology in permanent teeth, primarily for endodontic purposes, as detailed knowledge of apical anatomy is essentialis crucial for successful endodontic treatment [1, 2]. However, understanding age-related changes in apical anatomy is also crucial for evaluating healing processes following traumatic dental injuries.

A maxillary central incisor erupts at approximately 6 years of age, while root formation is completed about 6 years later [3]. In clinical practice, digital conventional periapical radiography is used to assess root development [3, 4].

It is well-known that teeth with completed root formation (mature teeth) are significantly more likely to develop a pulp necrosis (PN) than those with incomplete root formation (immature teeth) after a traumatic dental injury [5, 6]. This is because pulp revascularization depends on the diameter of the apical foramen [5–7]. The apical foramen (or major foramen) is the main apical opening of the root canal [8]. Andreasen et al. showed that a foramen diameter below 1.5 mm increases the risk of PN after extrusive and lateral luxation injuries, and intruded teeth with complete root development (≤ 0.7 mm) have a substantially higher PN risk than those with incomplete development (≥ 1.2 mm) [6]. The average size of the apical foramen in mature maxillary incisors has been found to be 0.4 mm by Green [9] and 0.297 mm by Chapman [10].

**Fig. 1:**
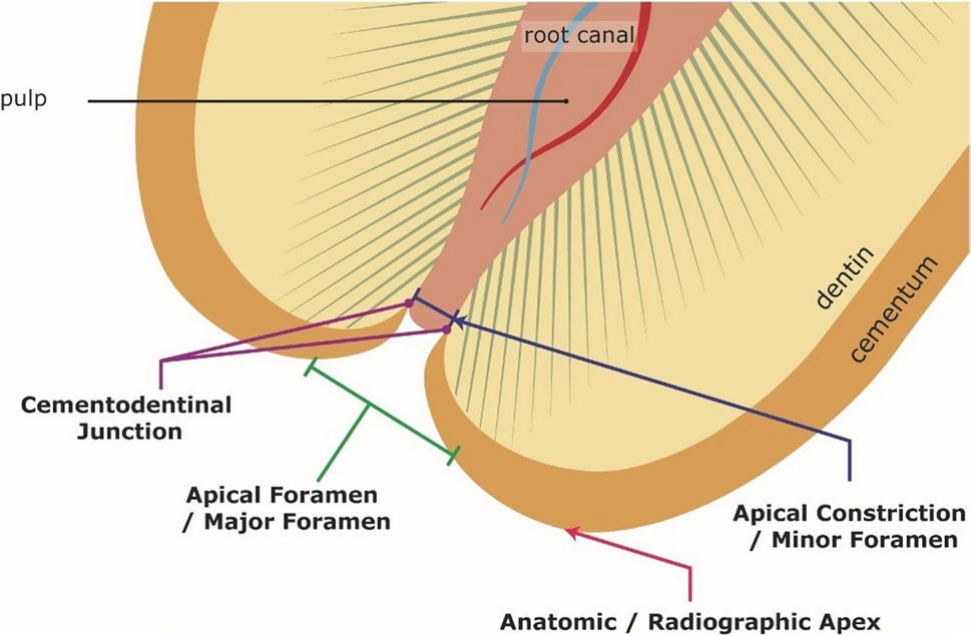
Anatomy of the apical region. (Adaptation from Kuttler [13] with permission)

The apical constriction (or minor foramen/minor diameter,) defined as the narrowest part of the canal near the apical foramen, is considered the ideal termination point for root canal preparation (Fig. 1) [11–14].

Kuttler investigated 268 teeth of all tooth types, mainly from cadavers, and divided the sample into two series: one series of teeth from individuals between 18 and 25 years of age (*N* = 142) and another from individuals 55 years of age or older (*N* = 126) [13]. Kuttler [13] reported the apical constriction to be located 0.524 mm from the foramen in individuals aged 18–25 years and 0.659 mm in those aged 55 years and above [13]. Similarly, Dummer et al. found a mean distance of 0.51 mm in 270 extracted human teeth (mandible and maxilla incisors, canines, premolars) in individuals of unknown age but with completely formed apices [12]. Chapman et al. revealed that the diameter of the apical constriction in maxillary incisors to be between 0.152 and 0.174 mm and between 0.135 and 0.140 mm for mandibular incisors [10]. In addition, Chapman et al. reported that 83% of the cases had an apical constriction that was circular shaped in cross-section. This study was based 120 extracted anterior (mandible and maxilla incisors and canines) human teeth of unknown age [10]. Table 1 summarizes some of the results from Chapman et al. [10], Kuttler [13] and Dummer et al. [12].

**Table 1:** Summarize of size, shape, tomography, distances and study material in apical region (Results from Chapman et al. [10], Kuttler [13] and Dummer et al. [12])

	Kuttler (1955) [13]	Chapman et al. (1969) [10]	Dummer et al. (1984) [12]
Study material	268 teeth from cadavers (all tooth types).	120 extracted anterior (mandible and maxilla incisors and canines) human teeth	270 teeth (mandible and maxilla incisors, canines, premolars) with completely formed apices
Age of study material	Two series: 18–25 (*N* = 142) years old and one from those 55 years and older (*N* = 126)	Unknown age	Unknown age
Size of apical constrictor	Apical constrictor located: - Cementum portion of the canal: 18–25 years: 0.255 mm 55 years and above: 0.240 mm - Dentinal portion of the canal; 18–25 years: 0.299 mm 55 years and above: 0.254 mm - Two even points CDC: 18–25 years: 0.244 mm 55 years and above: 0.210	Maxillary incisors: 0.152–0.174 mm. Mandibular incisors 0.135–0.140 mm	
Mean distance from apical constrictor to apical foramen	18–25 years: 0.524 mm. 55 years and above: 0.659 mm.	92.5% lie within 0.5–1.0 mm from the apex	0.51 mm
Diameter of foramen (average)	18–25 years: 0.502 mm. 55 years and above: 0.681 mm.	Maxillary teeth: 0.297 mm Mandibular teeth: 0.260 mm	
Mean distance from apical foramen to apex	18–25 years: 0.495 mm. 55 years and above: 0.607 mm	Maxillary teeth: 0.364 mm Mandibular teeth: 0.346 mm	Maxillary incisors: 0.23 mm ± 0.16 mm. Mandibular incisors: 0.36 mm ± 0.23 mm.
Most common shape of apical constrictor		Circular	
Tomography			Traditional single-constrictor form was most common.

Regarding the tomography(form) of the apikal constriction, the literature reports considerable variation [12, 15, 16]. Zhang et al. and Schell et al. identified the parallel form as the most common, whereas Dummer et al. reported the traditional single-constriction form [12, 15, 16].

To the best of our knowledge only a few studies have compared the apical anatomy in relation to the age of the patient [6, 13, 17]. Kuttler and Stein & Corcoran reported that the width of the major apical foramen increases with an increasing age due to the apposition of new cementum [13, 17].

Furthermore, Kuttler reported that the average apical constriction size varied with anatomical location and age [13]. Specifically, he found that in 26% of cases the minor diameter was found in the cementum portion of the canal (average diameter: 0.255 mm), 42% in the dentinal portion of the canal (average diameter: 0.299 mm) and 32% was found exactly at the height of the cemento-dentino-canal uniting point (CDC) (average diameter: 0.244 mm) in the 18- to 25-year-old patients [13]. Whereas for patients ≥ 55 years old, 29.5% the minor diameter was found in the cementum portion of the canal (average diameter: 0.240 mm), 29.5% in the dentinal portion of the canal (average diameter: 0.254 mm), and 41% was found exactly at the height of two even points CDC (average diameter: 0.210 mm) [13]. For each of the above-mentioned locations of the minor foramen, it is seen that the average diameter is smallest in the older patient group.

The aim of the present study was to investigate whether there is any discrepancy regarding the dimensions of the apical anatomy in maxillary incisors found on digital conventional periapical radiography and Cone-Beam CT scans (CBCT). Furthermore, to investigate whether the apical foramen narrows with increasing age. All the measurements were performed at narrowest part of the canal system (the apical constrictor/minor diameter).

## Materials and methods

### Material

The study followed the guidelines of the Declaration of Helsinki 2013 and a license to conduct the study and to store and handle the data was provided by the regional scientific ethical committee and the University of Osaka. The material was anonymized and consecutively collected during 2011–2023 at University of Osaka, Osaka, Japan as part of a local clinical standard routine. The majority of the CBCT images were obtained as part of orthodontic treatment protocol. In a few patients CBCT was obtained due to diagnostic purposes the in the presence of supernumerary teeth or lack of eruption of the canines.

Patients were included in the study based on the following inclusion criteria:

1. Presence of healthy, intact, and fully erupted permanent central maxillary incisor.
2. The stage of root development was defined as R_c_ and the apical closure A $\:\frac{1}{2}$ or A_c_ based on Moorrees classification [4].
3. Conventional periapical radiograph and CBCT scan including the maxillary central incisors taken not more than 30 days apart.
4. The quality of the images was good and without disturbing artifacts.

### Methods

For each tooth, the following parameters were measured.

#### Conventional periapical radiograph (2D)

Diameter of the apical foramen was measured in the mesiodistal direction on conventional periapikal 30,5 × 40,5 mm radiographs (ScanX Duo, ASAHIROENTGEN IND. CO., LTD. Kyoto) with high spatial resolution (968*1326).

The measurement was made at the narrowest part of the canal system (the apical constrictor) close to the apical foramen. The measurements were obtained between 0 and 1.0 mm from the apical foramen.

The intraoral x-ray images were calibrated in terms of geometrical dimensions by use of paired distance measurements carried out on well-defined global structures visible both in the x-rays and the CBCT images. The global structure chosen was the length of the tooth (from the incisal edge to the anatomic apex) that was measured in every tooth in the study in order to estimate a calibration factor.

The measurements were carried out using ImageJ (v1.54p, JAVA 1.8.0_345[64-bit]) [18].

#### Cone-beam computed tomography (CBCT, 3D)

The Cone-beam scanner employed was a CBCT-scanner Alphard VEGA (Alphard-3030, NEOPREMIUM, ASAHIROENTGEN IND. CO., LTD. Kyoto) acquiring images at 0.2 × 0.2 × 0.2 mm spatial resolution. The data were collected and evaluated using OnDemand3D^tm^ Dental (Ver. 1.0.10.7462).

At first, the topography of the apical part of the root canal was inspected – including longitudinal and cross sections of the tooth apex. To identify the apical constrictor of a root canal, the cross-section must be at the center of the canal to allow comparison of canal diameters and to locate the smallest one.

The size (diameter) of the apical foramen was measured in the mesiodistal and facio-lingual dimension in the axial slices/planes. This was performed by drawing a line between the two most distant pixels of the root canal walls at the narrowest part of the canal system (the apical constrictor) close to the apical foramen. The measurements were obtained between 0 and 0.60 mm from foramen. To get the best view of the relevant structures in the images the brightness and contrast were adjusted in the software.

In addition, the area of the foramen was measured in the axial plane. This was done by manually inserting points along the periphery/border of the foramen. A minimum of 10 points were inserted per measurement.

A single trained examiner (JSH) performed the 2D and 3D measurements. The examiner was a paediatric dentist trained by a senior researcher in paediatric dentistry with extensive experience in two- and three-dimensional medical imaging modalities. The examiner also had substantial experience working with 2D and 3D imaging in several previous research projects. Prior to the main analyses, a pilot sample was independently evaluated and subsequently discussed between the examiners to ensure consistency in assessment.

All measurements were repeated after two weeks in order to calculate intra-rater reliability. Bland‒Altman plots, Student’s t-test and linear regression were used to evaluate the agreement between the first and second measurement.

The Bland-Altman plots showed excellent agreement between first and second measurement of both faciolingual and mesiodistal diameter, and with bias close to zero (< 0.0002 mm), and with acceptable standard deviation of differences between the measurements (< 0.03 mm). The students’ t-test showed no significant difference between the first and second round of measurements (< 0.001). Pearson linear correlation coefficient R was 0.99 for the mesiodistal diameter and 1.00 for the facio-lingual diameter.

A shape index was defined to evaluate the shape of the apical foramen corresponding to the apical constriction. It was calculated using the following formula: $\:\frac{\mathrm{M}\mathrm{e}\mathrm{s}\mathrm{i}\mathrm{o}\mathrm{d}\mathrm{i}\mathrm{s}\mathrm{t}\mathrm{a}\mathrm{l}\:\mathrm{d}\mathrm{i}\mathrm{a}\mathrm{m}\mathrm{e}\mathrm{t}\mathrm{e}\mathrm{r}\:\:\mathrm{i}\mathrm{n}\:\mathrm{m}\mathrm{m}\:}{\mathrm{F}\mathrm{a}\mathrm{c}\mathrm{i}\mathrm{o}\mathrm{l}\mathrm{i}\mathrm{n}\mathrm{g}\mathrm{u}\mathrm{a}\mathrm{l}\:\mathrm{d}\mathrm{i}\mathrm{a}\mathrm{m}\mathrm{e}\mathrm{t}\mathrm{e}\mathrm{r}\:\:\mathrm{i}\mathrm{n}\:\mathrm{m}\mathrm{m}}$.

A comparison between the measurements of the apical foramen corresponding to the apical constriction in the mesiodistal dimension, determined on intraoral x-ray (2D) and CBCT (3D), respectively, was carried out using Student’s t test and linear regression.

A possible relation between the apical anatomy and age was investigated by regressing apical dimensions on age.

IBM SPSS Statistics (Version 29.0.1.0) and IDL (Interactive Data Language Version 9.0, ITT Visual Information Solutions) were used for statistics and data analysis. The analysis was performed by TAD.

**Fig. 2:**
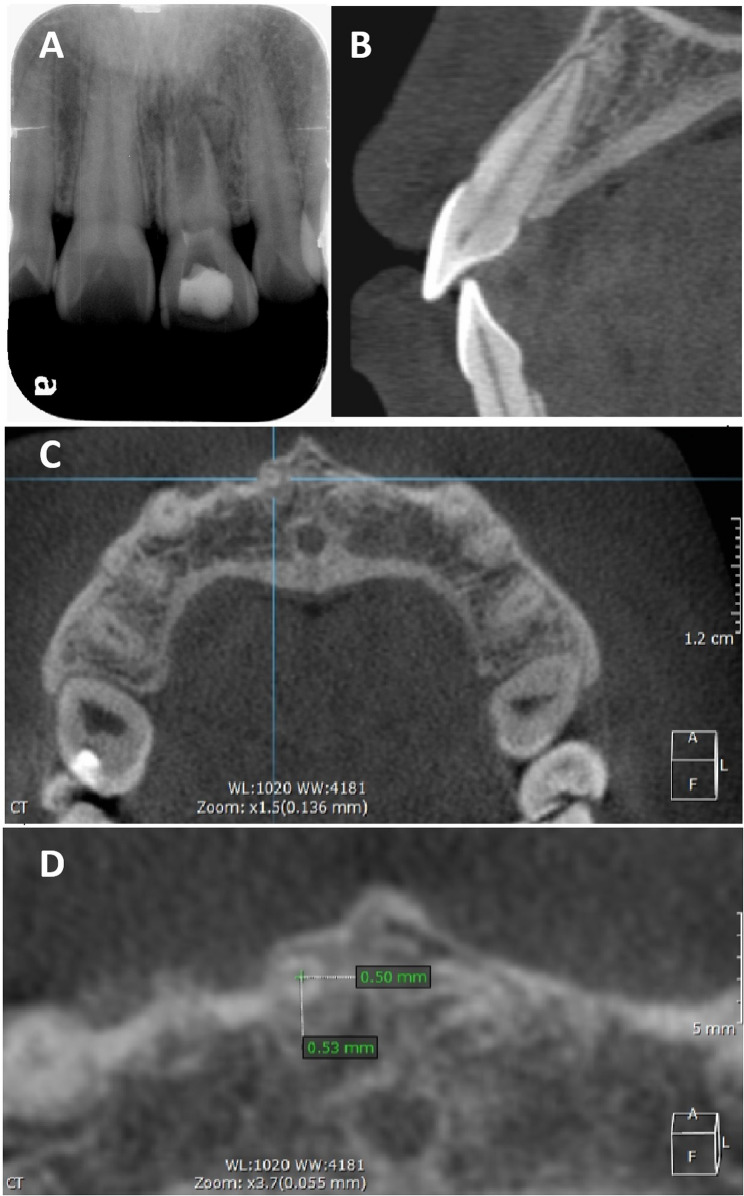
Representative case of tooth 11. The patient was 16 years old, and the tooth was classified as mature with apical closure. **A** shows the periapical radiograph, (**B**) a sagittal CBCT image view of the tooth, (**C**) an axial CBCT image view of the apical foramen and (**D**) a magnified axial CBCT image view showing measurement of the apical constrictor diameter in the mesiodistal and faciolingual directions

Level of significance was set at *P* = 0.05. A representative case is shown in Fig. 2.

## Results

A total of 70 patients with 87 teeth were included in the study (49 were male, 38 were female). The participants were between 7 and 39 years old (mean: 18.83 years, SD: 9.49). The apical constriction was on average located 0.27 mm from the foramen.

The correlation between mesiodistal diameter measured on the intraoral x-ray and CBCT was fairly strong with *R* = 0.60 (95% confidence interval [0.44,0.72]). The mean diameter of the foramen measured on intraoral periapical x-ray measured in the mesiodistal dimension were 0.52 mm ± 0.24, whereas it was 0.65 mm ± 0.21 on CBCT. The means were significantly different (P-value = < 0.0001). The size of the foramen was approximately 20% smaller when measured on intraoral x-rays compared to the CBCT scans.

The correlation between the mesiodistal and faciolingual diameter in CBCT wase strong with *R* = 0.77 (95% confidence interval [0.66, 0.84]).

The mean shape index was found to be 0.992 ± 0.244. This means that the present study found round to be the most common shape of the apical foramen corresponding to the apical constriction. However, a large variation is seen in the dataset. There was not a significant relation between the patients age and the shape of the apical anatomy (correlation coefficient was *R* = 0.01).

**Fig. 3:**
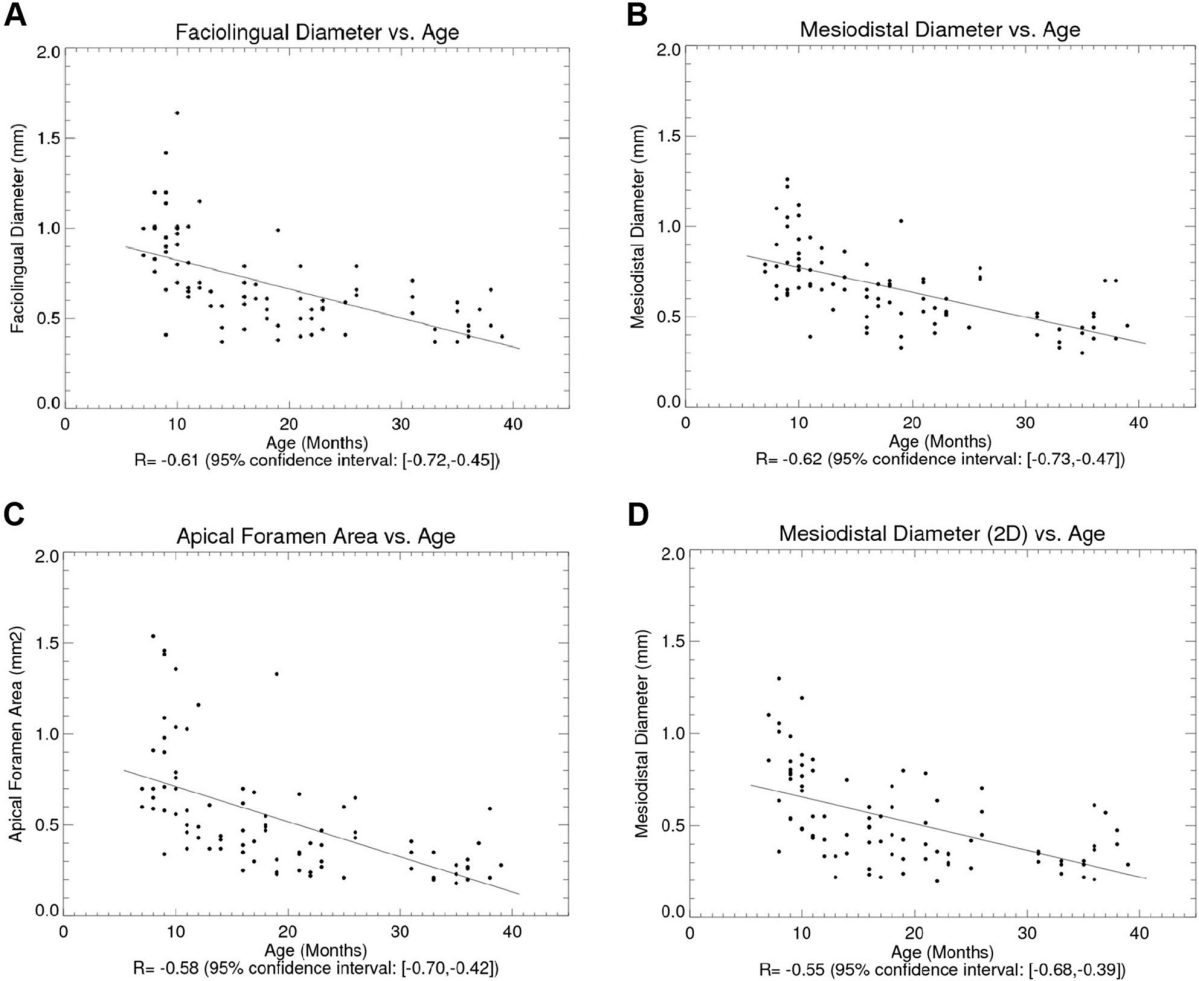
Correlation plots between age and diameter/area of ​​foramen measured on CBCT and Intraoral periapical x-ray. **A** illustrates the relationship between faciolingual diameter and age (measured on CBCT) (**B**) illustrates the relationship between mesiodistal diameter and age (measured on CBCT) (**C**) illustrates the relationship between apical foramen area and age (measured on CBCT) (**D**) illustrates the relationship between mesiodistal diameter and age (measured on Intraoral periapical x-ray)

The present study found that area of the foramen, measured at the level of the apical constrictor, decreased with increasing age. More specifically, it decreased at a rate of −0.019 mm_2_ per month. The mean area of the apical constrictor was found to be 0.72 mm for patients ≤ 15 years, 0.45 mm for patients 16–20 years, 0.34 mm for patients between 21 and 25 and 0.32 for patients above the age of 25 years of age. There is a particularly large variation in the size (area) of the apical foramen in the younger age groups. The older the patients, the smaller the variation. Gender did not affect the area significantly (Fig. 3; Table 2).

**Table 2:** Table of correlations/linear regressions with age, for the entities given R is Pearson correlation coefficient. Correlation is significant if 95% CI does not encompass 0.0. Slope is the a in the regression line given by y = ax+b

Type	*R*	95% CI	Slope
Faciolingual diameter vs. Age *(CBCT)*	−0.61	[−0.72, −0.45]	−0.016 mm/month
Mesiodistal diameter vs. Age *(CBCT)*	−0.62	[−0.73, −0.47]	−0.013 mm/month
Apical foramen area vs. Age *(CBCT)*	−0.58	[−0.70, −0.42]	−0.019 mm^2^/month
Mesiodistal diameter vs. Age *(Intraoral periapical x-ray)*	−0.55	[−0.68, −0.39]	−0.014 mm/month

The diameter of the apical constriction also decreased with increasing age irrespective of the radiographical method used and of the dimension (faciolingual or mesiodistal) measured. On the conventional periapikal radiographs the diameter of the foramen decreased with rate of −0.014 mm per month. On the CBCT scan the diameter of the foramen decreased with − 0.013 mm per month in the mesiodistal dimension, and − 0.016 mm per month in the facio-lingual dimension (Table 2). ANCOVA (analysis of covariance) was used in order to test for differences in slopes and y-intercepts of the regression lines. None of the three slopes (faciolingual diameter vs. age, mesiodistal diameter (CBCT) vs. age and mesiodistal diameter (*periapical radiograph)* vs. age) (in mm/month) had significantly different slopes (*p* < 0.05).

2D and CBCT regression lines (mesiodistal diameter vs. age) have significantly different y-intercepts (measured Apical Foramen Area values are smaller in 2D than in CBCT).

## Discussion

Our hypothesis was that there is a discrepancy regarding the size of the apical foramen as seen in conventional periapical radiographs (two-dimensional) and CBCT scans (three-dimensional). The shape of the apical foramen was only evaluated on CBCT scan; however, we hypothesized that the most common shape was oval. Furthermore, it was hypothesized that even though the maxillary incisor appears with a closed apex on the conventional periapical radiograph (and therefore traditionally will be classified to be mature), there were still some ongoing changes in the apical area. And finally, that the apical foramen, measured at narrowest part of the canal system (the apical constrictor), narrows with increasing age.

Our hypothesis regarding the shape of the apical constriction was rejected. However, we did not reject our hypothesis on a discrepancy in foramen size measurements between periapical radiographs (two-dimensional) and CBCT scans (three-dimensional). Furthermore, we did not reject our hypothesis on although the apical foramen appears closed on conventional periapical radiographs, there are still ongoing changes in the apical area and that the apical constriction narrows with increasing age.

This study found round to be the most common shape of the apikal constriction, while oval foramina were evenly distributed between the faciolingual and mesiodistal directions. Similar results are reported by Chapman who found a circular apical constriction in 83% of cases showed [10].

For comparison, several studies have described the shape of the apical foramen in maxillary central incisors, however, no consensus has been reached. Swathika et al. examined extracted permanent mature teeth in a stereomicroscope found the most common shape of the apical foramen in maxillary central incisors was oval (56.67%) followed by round (40%) and uneven shape (3.33%) [1]. Manva et al. reported similar results (oval shape: 55%) [19]. On the other hand, Martos et al. reported that round was the most common shape [20]. Importantly, it should be recognized that the apical foramen is not uniformly round, and assuming otherwise may lead to misinterpretation, particularly when assessed in two dimensions. For example, when an oval foramen is elongated in the faciolingual direction, may appear more constricted (closed) on periapical radiographs than if analyzed in three dimensions.

An interesting finding from this present study is that the area of the apical constrictor appears to vary more in younger patients than adults. A plausible explanation for this could be that even though the tooth is categorized as mature (the root development seems completed) in a young patient, there is still a significant deposition of dentin going on apically, which causes the apical area to become even more narrow.

**Fig. 4:**
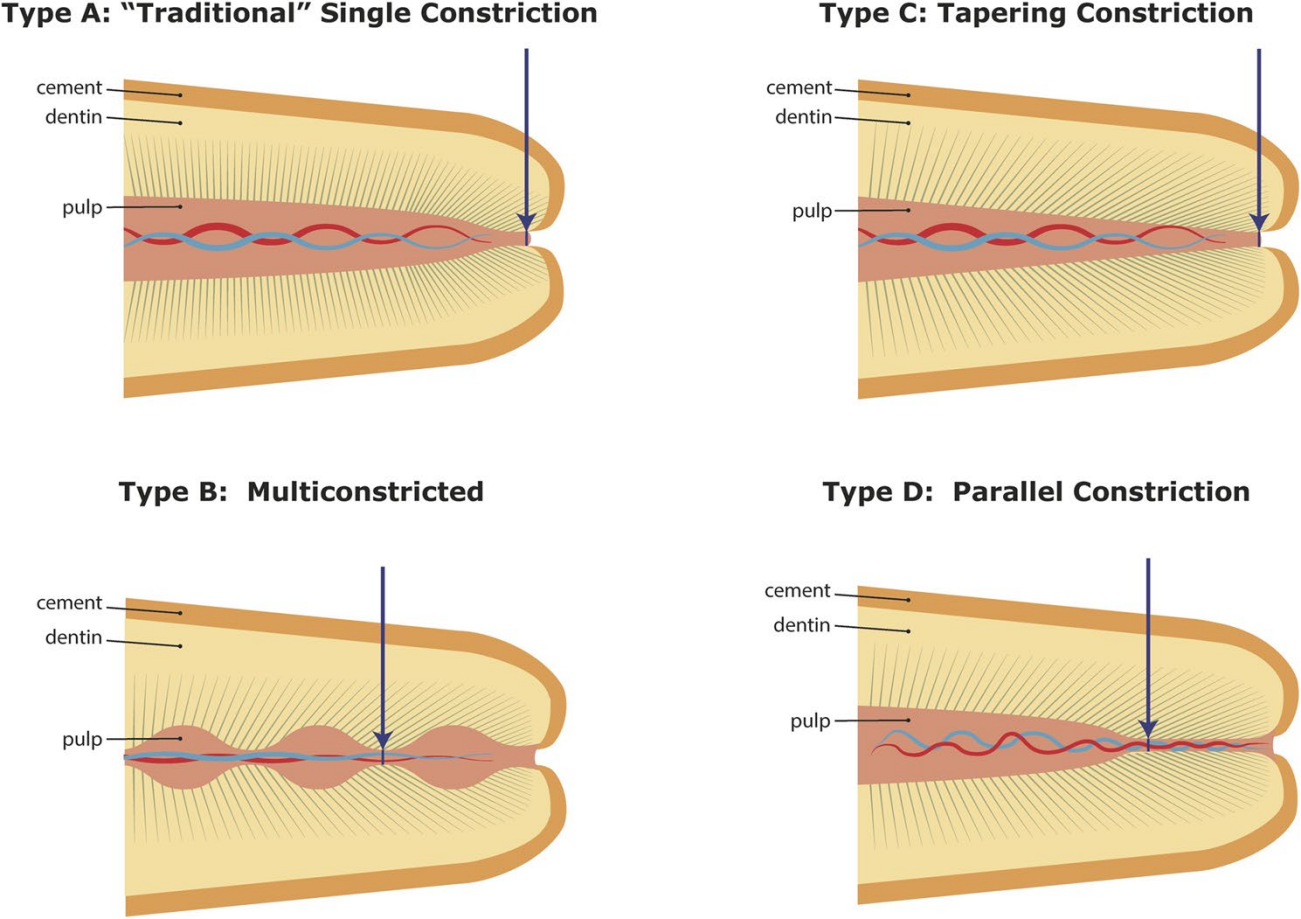
Types of apical constriction (Adaptation from Dummer et al. [12] with permission)

The topography of the apical constriction can also vary [12, 16]. In 1984 Dummer et al. found that the topography of the apical constriction was not constant. The study was based on examination of longitudinal sections of the root. This led to the classification of 4 different apical constriction types [12]. See Fig. 4. However, Schell et al. questioned the method (the use of longitudinal views of the canal). The study compared longitudinal sections with cross sections for disclosing the topography and location of the apical constriction [16]. The study concluded that cross-sectional analysis of the topography of the apical constriction provides a more consistent information about the existence, topography, and location of the apical constriction than longitudinal Sect [16]. Canal curvatures and deviations of the shape of the apical constrictor can explain why longitudinal sections may not be a suitable method. In the present study, the examination of the apical constriction was made in the axial slices/plane from cross-sections, thereby the tomography was not analyzed.

The sample of this study included central maxillary incisors, making it highly comparable to those of Chapman and Dummer et al. [10, 12]. A notable limitation of both studies, however, was that the age of the samples was not reported. In contrast, Kuttler considered age, yet his findings were not stratified by individual tooth types [13].

The apical constriction was on average located 0.27 mm from the foramen, slightly lower but still comparable with previous reports. A key difference, however, is that Kuttler’s results represented an average across all tooth types [13], and Dummer et al. also included canines and premolars [12], whereas the present study focused exclusively on central maxillary incisors. This may explain the variation and underlines the importance of tooth type–specific analyses.

Without correcting for age, the present study found a mean mesio-distal diameter of the apical constrictor to be 0.65 mm on CBCT. The discrepancy between this value and that reported by Chapman may possibly be explained by differences in the age of the samples; however, this remains speculative and cannot be confirmed [10]. Notably, Kuttler demonstrated that the average diameter of the apical constriction varied with both its location and the age of the patients, with the smallest mean diameter of the minor foramen observed in older individuals [13]. This age-related trend aligns with the present findings and supports the importance of considering patient age when interpreting apical morphology.

Such age-related differences in apical dimensions are closely associated with well-documented physiological changes in the dentin–pulp complex. Secondary dentin synthesis and apposition begins when root formation is completed and throughout life. In maxillary incisors it is mainly deposited on the walls of the pulp chamber [21–23]. Whereas secondary dentinogenesis represents a normal and expected physiological process occurring with aging, tertiary dentin formation is deposited in response to injury or trauma leading to calcification in the pulp lumen and root canal system. These events may lead to a substantial reduction of the pulp and root canal lumen over time [21–23]. Studies found that 90% of the individuals over 40 years had some degree of calcification of the pulp lumen that leads to obliteration of the canal [24, 25]. The participants in this study were between 7 and 39 years old (mean: 18.83 years, SD: 9.49), however, it may be of interest to investigate this narrowing beyond the age range included in the present study to determine when, and whether, the age-related constriction reaches a plateau or stabilizes.

Other pathological events, like internal or external resorption, can also occur in the apical area. Zhang et al., found that anterior teeth with apical periapical inflammation had a larger apical constriction than healthy teeth presumably due to destruction in the area preceded by the periapical inflammatory process [15].

In general, this study’s trained examiner found that the measurements were much easier to perform on the CBCT scans compared with the periapical radiographs. A periapical radiograph represents a two-dimensional projection of a three-dimensional structure. Deviation of the main apical foramen from the anatomical apex is well-known [1, 9, 26], which complicates accurate measurements and is likely to introduce errors. Furthermore, distortion and superimposition of surrounding structures, such as bone and soft tissue, create additional challenges that may lead to misinterpretation and measurement inaccuracy. These limitations are avoided when using CBCT. In addition, CBCT provides high spatial resolution, and the 3D software enables the tooth to be angled, allowing the measurement to be made perpendicular to the foramen. In 1977 the International Commission on Radiologic Protection introduced the “As Low As Reasonably Achievable” (ALARA) principle [27]. Briefly, any radiation exposures should be justified, the exposure should be as low as reasonably achievable while still maintaining a suitable image quality and below the allowable limits [27]. Although this study indicates that CBCT offers a more accurate assessment of the apical anatomy, it does not seem justified to perform CBCT as part of standard procedure when determining the development of the root. Nevertheless, it is important to recognize that the apical foramen is most likely slightly larger than it appears on the intraoral radiographs.

A recent retrospective study reported a potential for pulp revascularization in mature anterior teeth with lateral luxation in patients up to 25 years of age [28]. The study further observed that the risk of PN increased with age. One of the suggested explanations behind this significant development, were that children and adolescents may have better healing potential than adults, as the apical foramen is not completely constricted, despite the root appearing fully developed (with a closed apical foramen) on a periapical radiograph [28]. The hypothesis was that the apical foramens narrowest point, the apical constriction, will decrease with increasing age. When determining the healing potential, the chance of pulp revascularized, after a traumatic tooth injury the stage of the root development is a key factor then deciding treatment. The findings of the present study are consistent with those of Henriksen et al. [29], including the proposed underlying explanation, namely, that age is a decisive factor [28]. The present findings may provide clinicians with a more nuanced understanding of apical anatomy that can serve as a reference in clinical decision-making. Consequently, treatment guidelines based solely on whether the tooth appears mature on a two-dimensional radiograph should not be recommended as exemplified by current international guidelines for treatment and management of a permanent mature laterally luxated tooth [29].

Clinically, this is relevant to keep in mind, as the shape cannot be decided from two-dimensional radiograph. Also, it is noteworthy that we found a size difference of about 20% which may be considered clinically relevant.

This study has several strengths. Unlike most previous investigations on extracted teeth, it examined apical anatomy in situ, providing clinically relevant data. The paired design with both periapical radiographs and CBCT of the same teeth allowed a direct comparison of imaging modalities, and the relatively broad age range offered insight into developmental changes beyond root completion. High intra-rater reliability further supports the validity of the measurements.

Nevertheless, some limitations must be acknowledged. Although the age range was broader than in many earlier studies, it did not include older adults in whom further narrowing may occur. Although CBCT provides high spatial resolution, precise definition of the apical foramen borders at the pixel level remains challenging and may introduce minor measurement errors. Comparable limitations are seen in conventional radiographs, where resolution limits and structural superimposition hinder reliable identification of apical anatomy. In addition, inter-rater reliability was not evaluated, and future studies should include multiple observers to confirm the reproducibility of the findings.

Future studies should include larger, tooth type - specific samples across wider age ranges. Combining cross-sectional and longitudinal approaches may provide a more comprehensive understanding of apical anatomy.

## Conclusion

The present study finds that area and the diameter of the apical foramen, measured to be equivalent to the apical constrictor, decreased with increasing age even though root development of all included teeth were classified as mature.

There is a significant difference between the diameter of the foramen measured on conventional periapical radiographs compared to the CBCT scans. The measurements are around 20% smaller on periapical radiographs.

Concerning the apical constriction shape, round is the most common shape observed in our sample.

## Data Availability

The datasets used and/or analyzed during the current study are available from the corresponding author on reasonable request.

## Abbreviations

CBCT: Cone-beam computed tomography
CDC: Cemento-dentino-canal uniting point
PN: Pulpa necrosis
